# Inhibiting Extracellular Cathepsin D Reduces Hepatic Steatosis in Sprague–Dawley Rats †

**DOI:** 10.3390/biom9050171

**Published:** 2019-05-04

**Authors:** Princy Khurana, Tulasi Yadati, Sandeep Goyal, Atul Dolas, Tom Houben, Yvonne Oligschlaeger, Anil K. Agarwal, Aditya Kulkarni, Ronit Shiri-Sverdlov

**Affiliations:** 1 Aten Porus Lifesciences Pvt Ltd., Bengaluru, Karnataka 560068, India; princy@atenporus.com (P.K.); sandeep@atenporus.com (S.G.); atul@atenporus.com (A.D.); aditya@atenporus.com (A.K.); 2 Department of Molecular Genetics, School of Nutrition and Translational Research in Metabolism (NUTRIM), Maastricht University, 6229 ER Maastricht, The Netherlands; t.yadati@maastrichtuniversity.nl (T.Y.); tom.houben@maastrichtuniversity.nl (T.H.); y.oligschlaeger@maastrichtuniversity.nl (Y.O.); 3 Department of Chemistry, CHRIST (Deemed to be University), Bengaluru, Karnataka 560029, India; anilkumar.agarwal@christuniversity.in; 4 Avaliv Therapeutics, Naples, FL 34120, USA

**Keywords:** NAFLD, lysosomal enzyme, extracellular cathepsin D, small-molecule inhibitor

## Abstract

Dietary and lifestyle changes are leading to an increased occurrence of non-alcoholic fatty liver disease (NAFLD). Using a hyperlipidemic murine model for non-alcoholic steatohepatitis (NASH), we have previously demonstrated that the lysosomal protease cathepsin D (CTSD) is involved with lipid dysregulation and inflammation. However, despite identifying CTSD as a major player in NAFLD pathogenesis, the specific role of extracellular CTSD in NAFLD has not yet been investigated. Given that inhibition of intracellular CTSD is highly unfavorable due to its fundamental physiological function, we here investigated the impact of a highly specific and potent small-molecule inhibitor of extracellular CTSD (CTD-002) in the context of NAFLD. Treatment of bone marrow-derived macrophages with CTD-002, and incubation of hepatic HepG2 cells with a conditioned medium derived from CTD-002-treated macrophages, resulted in reduced levels of inflammation and improved cholesterol metabolism. Treatment with CTD-002 improved hepatic steatosis in high fat diet-fed rats. Additionally, plasma levels of insulin and hepatic transaminases were significantly reduced upon CTD-002 administration. Collectively, our findings demonstrate for the first time that modulation of extracellular CTSD can serve as a novel therapeutic modality for NAFLD.

## 1. Introduction

Non-alcoholic fatty liver disease (NAFLD) is one of the most common liver diseases in the world with an estimated prevalence of 25% in the adult population [1]. Histologically, NAFLD ranges from steatosis, which is the benign accumulation of macro-vesicular and micro-vesicular lipid droplets in hepatocytes, to the more severe form of NAFLD referred to as non-alcoholic steatohepatitis (NASH). NASH is a combination of hepatic steatosis and inflammation with or without fibrosis [2], which can progress further to severe conditions such as liver cirrhosis and liver failure [3,4]. Due to the lack of mechanistic insights into the pathogenesis of NAFLD [5], clinically-approved treatment options do not yet exist.

NAFLD is characterized by an impaired hepatic lipid profile, which is in turn characterized by dysregulation of cholesterol and triglyceride metabolism [6]. It has been well-established that lysosomes, in particular their lysosomal proteases such as cathepsins, play a crucial role in maintaining metabolic processes such as lipid homeostasis [7,8,9,10]. Specifically, it has been shown that changes in the expression of the lysosomal enzyme cathepsin D (CTSD) are associated with differences in cholesterol metabolism [11,12,13]. Relevantly, though CTSD is mainly functional within the intracellular, acidic milieu of lysosomes, in response to certain physiological and pathological conditions, CTSD is secreted extracellularly [11]. Strikingly, and similar to its intracellular fraction, it has been shown that CTSD is also proteolytically active upon secretion into the extracellular space [14,15]. Indeed, given that CTSD retains its catalytic activity at neutral pH [16], these data indicate a potential role for extracellular CTSD in lipid-related disorders such as NAFLD [17,18]. 

Recently, we have demonstrated that targeting the proteolytic activity of CTSD using pepstatin-A (Pep-A), a known inhibitor of CTSD, remarkably reduced steatohepatitis in a hyperlipidemic mouse model, thereby pointing towards a role for CTSD activity in NAFLD [19]. However, the observed effects in this study were the result of a targeted inhibition of intracellular and extracellular CTSD. Moreover, Pep-A is a potent but relatively unspecific inhibitor of aspartic proteases [20]. Hence, in the current study, we developed a highly specific and potent small-molecule inhibitor of extracellular CTSD, referred to as CTD-002 (IC50 = 28 nM) in order to investigate its efficacy in treating NAFLD.

We hypothesized that specific inhibition of extracellular CTSD activity has a therapeutic benefit for NAFLD. In order to test our hypothesis, Sprague–Dawley (SD) rats fed a high-fat diet (HFD), known as the Lieber–Decarli diet, were used as an in vivo NAFLD model [21] and were injected with the CTD-002. In addition, wild-type (Wt) bone marrow-derived macrophages (BMDMs) treated with CTD-002 were used as an in vitro model to assess the ability of CTD-002 to reduce inflammation. Moreover, to investigate whether the CTD-002 inhibitor influences the inflammatory crosstalk between hepatic immune and parenchymal cells, a conditioned medium derived from BMDMs was transferred to HepG2 liver cells. Treating BMDMs with CTD-002 resulted in a decrease in inflammation and improvement in lipid metabolism. In addition, incubating hepatic HepG2 cells with the conditioned medium derived from CTD-002-treated BMDMs showed a decreasing trend in inflammation compared to control cells. Sprague–Dawley rats fed an HFD showed decreased hepatic steatosis and inflammation upon treatment with CTD-002. Altogether, these data suggest that targeting extracellular CTSD potentially represents a novel and effective therapeutic strategy for NAFLD.

## 2. Materials and Methods

### 2.1. Design and Development of CTD-002

CTD-002 was designed using the Schrodinger Small Molecule Drug Design Suite and medicinal chemistry efforts. In this process, a library of ~500,000 commercially available compounds were screened in silico using the pharmacophore modelling [22,23] in PHASE module and docking the top 10,000 molecules via the GLIDE XP [24,25] module of the Schrodinger suite (v7.2, Schrödinger, LLC). This resulted in the identification of a set of 100 molecules with diverse chemical structures. These molecules were then further screened against CTSD in a cell-free assay to identify those compounds that had micromolar or higher potency. The compounds with the lowest permeability and efflux were selected as exerting most of its effects extracellularly. Finally, these compounds were further refined and modified to create a library of compounds with nanomolar efficacy against CTSD, with CTD-002 being the most potent small-molecule inhibitor, showing a dose-dependent inhibition of CTSD activity (Figure S1). While the compound may have minor effects of intracellular CTSD, toxicity studies in Sprague–Dawley rats and BALB/c mice, a widely used mouse strain, showed no toxic reactions at the therapeutic dose of 50 mg/kg, which was injected twice a week for six weeks.

### 2.2. Isolation and Culturing of Bone Marrow-Derived Macrophages

Tibiae and femurs of wild type mice belonging to C75Bl/6 strain were broken and used for harvesting bone marrow-derived macrophages (BMDMs). The culture medium in which the BMDMs were cultured was an RPMI medium (GIBCO Invitrogen, Breda, the Netherlands) supplemented with 10% heat-inactivated fetal calf serum (Bodinco B.V. Alkmaar, the Netherlands), penicillin (100 U/mL), streptomycin (100 μg/mL) and L-glutamine 2 mM (all derived from GIBCO Invitrogen, Breda, The Netherlands). To differentiate between monocytes and macrophages, the culture medium was also supplemented with 20% L929-conditioned medium (LCM) for 9 days. At day 10, BMDMs were cultured in a 24-well plate at 350,000 cells per well. Next, the BMDMs’ culture medium was enriched with oxidized low-density lipoprotein (oxLDL) for 24 h (25 μg/mL; Alfa Aesar: J65591, Wardhill, MA, USA). Then, the macrophages were treated with CTD-002 (Aten Porus Lifesciences Pvt Ltd., India) or with carrier-control dimethyl sulphoxide (DMSO) for 4 h. In order to increase the effects on inflammation, cells were washed and further stimulated with lipopolysaccharide (LPS; 100 ng/mL) for 4 h. 

To explore the possible effects of secreted CTSD on neighboring cells, hepatic HepG2 (ATCC HB-8065) cells were incubated with the conditioned medium from CTD-002-treated BMDMs, or control-treated BMDMs, for 4 h. Subsequently, cells were washed and stimulated with LPS (100 ng/mL) for 4 h, after which the supernatant was collected for protein measurements and cells were lysed for mRNA expression analyses.

### 2.3. Rats, Diet and Intervention

Animal experiments were conducted at TheraIndx Lifesciences Private Limited, following all ethical practices as formulated in the guidelines for animal care and approved by the Institutional Animals Ethics Committee (IAEC; protocol no. IAEC/05/2017/064), India. SD rats were given free access to food and water and were housed under standard conditions. Following an acclimatization period of seven days, rats were fed with either low-fat (LFD) or HFD for three weeks. The composition of the diets is listed in Table S1. To test its therapeutic efficacy, CTD-002 was administered twice a week intraperitoneally (dose 50 mg/kg) for three weeks. The experimental conditions are also shown in Figure S2. After three weeks, the animals were sacrificed using CO2 anesthesia. Following blood collection, the rats were dissected, liver tissues were excised, weighed and cut into small pieces weighing ~100 to 200 mg. These liver tissues were frozen immediately in liquid nitrogen and stored at −80 °C for further analyses. In addition, liver samples were also fixed in 4% formaldehyde for histologic examination. Blood was collected by cardiac puncture followed by termination, centrifuged and plasma was harvested for biochemical analyses.

### 2.4. Histological Analyses

Formalin-fixed liver samples were stained with Hematoxylin-Eosin (H&E) in order to score fat vesicles as minimal, mild, moderate and severe steatosis. Lobular inflammatory activity was scored as follows: (1) focal collections of mononuclear inflammatory cells; (2) diffuse infiltrates of mononuclear inflammatory cells and (3) focal collections of polymorphonuclear cells in addition to mononuclear cell infiltrates. In addition to H&E, formalin-fixed liver samples stained with Chromotrope Aniline Blue and Sirius Red were quantified for total hepatic fat percentage by means of digital image analysis in ImageJ. 

### 2.5. Plasma Measurements

Plasma concentrations of alanine aminotransferase (ALT) [S.G.P.T ERBA kit, 120903; Baddi, India] and aspartate aminotransferase (AST) [S.G.O.T ERBA kit, B081717; Baddi, India] were measured with an enzymatic color test according to the manufacturer’s protocols and were measured using an ERBA semi-automated biochemistry analyzer. Plasma insulin was measured using a rat enzyme-linked immunosorbent assay kit [ab100578, Abcam; Cambridge, MA, USA]. 

### 2.6. Liver Tumor Necrosis Factor-Alpha Levels

The rat tumor necrosis factor-alpha (TNF-α) ELISA assay was performed on liver homogenates according to the manufacturer’s instructions [ab100785, Abcam; Cambridge, MA, USA] using a microplate reader. 

### 2.7. Statistical Analyses

Data were statistically analyzed by performing two-tailed non-paired *t*-tests using GraphPad Prism, version 6.0 for Windows. Data were expressed as mean ± SEM and considered significant at *p* < 0.05. *, ** and *** indicate *p* < 0.05, 0.01 and 0.001, respectively.

## 3. Results

### 3.1. Decreased Inflammation after Inhibition of Extracellular CTSD in oxLDL-Loaded Primary Mouse Macrophages

**Figure 1:**
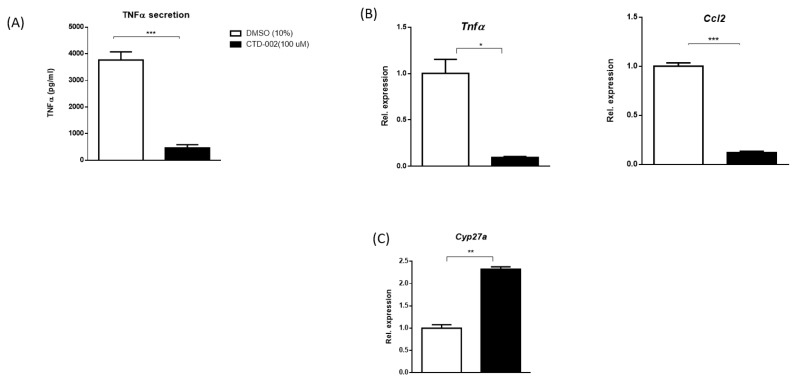
Effects of CTD-002 on inflammation and cholesterol metabolism in bone marrow-derived macrophages. (**A**) TNFα protein levels measured from supernatant of oxidized low-density lipoprotein (oxLDL)-loaded bone marrow-derived macrophages (BMDMs). (**B**) Gene expression of inflammation-related genes *Tnfα* and *Ccl2* and (**C**) the cholesterol breakdown enzyme *Cyp27a1* in oxLDL-loaded bone marrow-derived macrophages. Each bar represents a technical triplicate ± SEM; * means *p* < 0.05, ** *p* < 0.01 and *** *p* < 0.001 compared to dimethyl sulfoxide (DMSO)-treated BMDMs by means of two-tailed unpaired *t*-test.

With the aim of exploring the influence of extracellular CTSD on inflammation in macrophages and to test the efficacy of the small-molecule inhibitor CTD-002, Wt BMDMs were isolated and incubated with oxLDL for 24 h to stimulate the secretion of CTSD. Subsequently, cells were treated with either carrier control (DMSO) or CTD-002 (100 µM) for 4 h, followed by 4 h stimulation with LPS. Tumor necrosis factor α (TNFα) protein levels, a pro-inflammatory cytokine, measured from the medium of CTD-002-treated BMDMs, were significantly reduced compared to control cells (Figure 1A). Consistent with these results, gene expression levels of the pro-inflammatory cytokines *Tnfα* and chemokine (C-X-C motif) ligand-2 (*Ccl2*) significantly decreased in CTD-002-treated macrophages compared to control (DMSO)-treated cells (Figure 1B), confirming the reduction of inflammation upon treatment with CTD-002. Additionally, extracellular CTSD inhibition also improved cholesterol metabolism, as evidenced by the upregulation of cytochrome P450 27a1 (*Cyp27a*)1, the main enzyme responsible for the conversion of cholesterol into bile acids (Figure 1C). Similar data were also observed in control conditions without oxLDL and LPS stimulation, showing reduced *Tnfα* and *Ccl2* levels and improved lipid and energy metabolism as evidenced by *Cyp27a1,* acetyl-CoA acetyltransferase 2 (*Acat2),* carnitine palmitoyltransferase1 *(Cpt1)* and scavenger receptor A (*Sr-a*) expression after CTD-002 treatment (Figure S3). Collectively, these data indicate that modulating the activity of extracellular CTSD has beneficial effects on inflammation and cholesterol metabolism, at least in vitro.

### 3.2. Inhibition of Macrophage-Derived Extracellular CTSD Reduces Inflammation in HepG2 Cells

**Figure 2:**
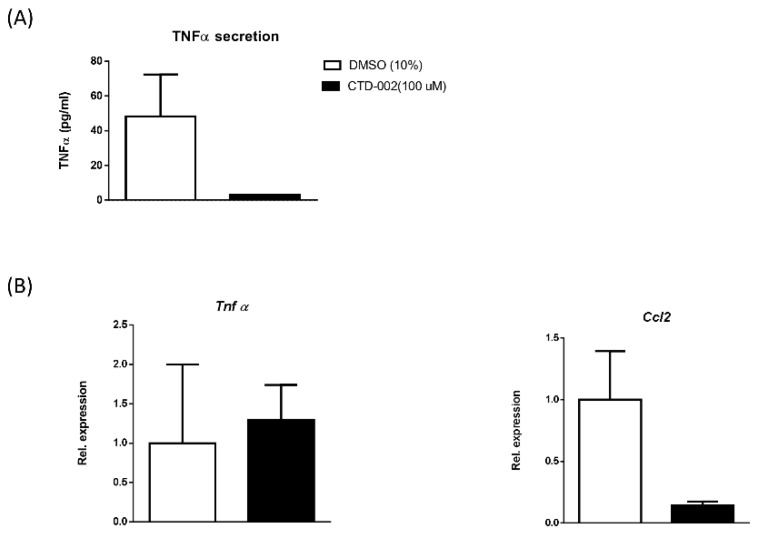
Effect of medium derived from CTD-002-treated BMDMs on HepG2 cells. (**A**) TNFα cytokine secretion of HepG2 cells cultured in a macrophage-conditioned medium that was treated with or without CTD-002. (**B**) Gene expression levels of *Tnfα* and *Ccl2* measured in HepG2 cells. Error bars represent mean ± SEM.

In order to confirm whether macrophage-derived extracellular CTSD influences neighboring parenchymal cells, hepatic HepG2 cells were incubated with a conditioned medium derived from macrophages that were treated either with carrier-control or with CTD-002 (100 µM) for 4 h. To boost the effects on inflammation, HepG2 cells received LPS for 4 h. HepG2 cells that received the conditioned medium from macrophages that were treated with CTD-002 showed a trend towards reduced TNFα secretion (Figure 2A). Additionally, HepG2 gene expression levels of *Ccl2* showed a decreasing trend compared to HepG2 cells that were incubated in the control-treated conditioned medium, while *Tnfα* did not show any effect (Figure 2B). Additionally, we also observed a reduced expression of the cluster of differentiation 36 (*Cd36*) in hepatocytes receiving the conditioned medium that was derived from BMDMs treated with CTD-002, suggesting an effect on hepatocyte lipid metabolism (Figure S4). These in vitro findings suggest that macrophage-derived extracellular CTSD affects the inflammatory status of neighboring parenchymal cells and that inhibiting extracellular CTSD activity reduces this inflammatory response.

### 3.3. Improved Metabolic Features after Inhibition of Extracellular CTSD in HFD-Fed Sprague–Dawley Rats

**Figure 3:**
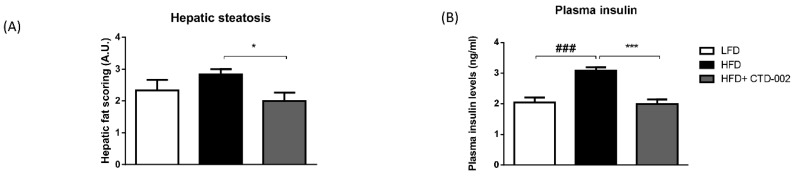
Metabolic parameters of high-fat diet (HFD)-fed Sprague–Dawley (SD) rats treated with or without the extracellular inhibitor CTD-002. (**A**) Scoring of hepatic steatosis by means of Hematoxylin-Eosin (H&E) staining. (**B**) Plasma levels of insulin. Error bars represent mean ± SEM; *n* = 6 for each group; * represents *p* < 0.05 and *** *p* < 0.001 compared to rats on HFD; ### represents *p* < 0.001 compared to the rats on low-fat diet (LFD) by means of two-tailed unpaired *t*-test.

To elucidate the metabolic effects of extracellular CTSD inhibition in vivo, metabolic parameters were tested in HFD-fed SD rats that were injected with or without the CTD-002 inhibitor. Though food consumption was statistically similar among all experimental groups, a trend towards reduced food intake was apparent (Figure S5). By performing an H&E staining, hepatic steatosis was assessed based on the scoring of fat vesicles in the liver (Figure S6). The impact of HFD CTD-002-treated HFD-fed SD rats showed a significant reduction in the amount of fat vesicles relative to control HFD-fed rats, demonstrating the therapeutic benefit of extracellular CTSD inhibition in the context of hepatic steatosis, a main feature of NAFLD (Figure 3A). The impact of the HFD on hepatic fat deposition was further confirmed by quantification of H&E, Chromotrope Aniline Blue and Sirius Red staining, showing increased fat deposition with the HFD (Figure S7). 

To further assess whether inhibition of extracellular CTSD influences pathological parameters related to NAFLD, plasma insulin levels were measured as an indicator of insulin sensitivity. Plasma insulin levels were significantly increased in HFD-fed SD rats compared to LFD-fed rats (Figure 3B). Further, CTD-002-treated HFD-fed rats had significantly lower plasma insulin levels compared to HFD-fed rats (Figure 3B). This finding suggests an improvement in insulin sensitivity, further emphasizing the metabolic benefit induced by inhibition of extracellular CTSD. Altogether, these data indicate that modulating extracellular CTSD activity improves metabolic features associated with NAFLD. 

### 3.4. Reduced Liver Damage after Inhibition of Extracellular CTSD in HFD-Fed Sprague–Dawley Rats

**Figure 4:**
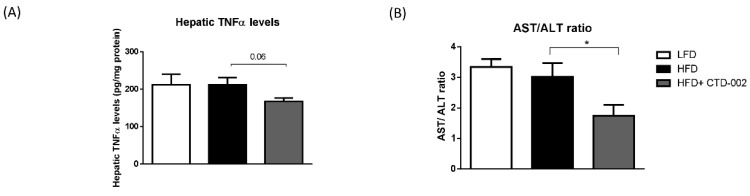
Hepatic TNFα levels and aspartate transaminase/alanine transaminase (AST/ALT) ratio in HFD-fed SD rats treated with or without the extracellular inhibitor of cathepsin D (CTSD). (**A**) Hepatic TNFα levels. (**B**) Plasma transaminase levels. Error bars represent mean ± SEM; *n* = 6 for each group. * indicates *p* < 0.05 compared to rats on the HFD.

To determine the effects of extracellular CTSD inhibition on hepatic inflammation, the protein levels of hepatic TNFα were measured in HFD-fed SD rats that were treated with or without the CTD-002 inhibitor of extracellular CTSD. CTD-002-treated HFD-fed SD rats showed a trend towards reduced hepatic TNFα levels (*p* = 0.06) compared to the HFD-fed group (Figure 4A), thereby suggesting an improvement in hepatic inflammation. To further assess the effects of extracellular CTSD inhibition on liver damage, plasma aspartate aminotransferase (AST) and alanine aminotransferase (ALT) levels were measured. Upon treatment with CTD-002, HFD-fed SD rats showed a significant reduction in the aspartate transaminase/alanine transaminase (AST/ALT) ratio (Figure 4B), pointing towards an improvement in liver damage.

## 4. Discussion

Treating NAFLD is of critical importance to prevent further progression to liver cirrhosis and end-stage liver disease. The present study provides the first evidence that modulating extracellular CTSD activity can significantly improve metabolic parameters associated with NAFLD. These findings suggest the potential therapeutic benefits of targeting extracellular CTSD within the context of metabolic diseases and opens new perspectives for therapeutic interventions in NAFLD.

To elucidate the effects of extracellular CTSD inhibition in NAFLD, we have used SD rats that were fed a high-fat diet for three weeks. Sprague–Dawley rats are known to develop micro-vesicular steatosis with [21] or without inflammation [26], depending on the composition and duration of the HFD. Here, SD rats developed hepatic steatosis. Similar to the findings of Lieber and Ahmed et al. [21,26], no changes in relative liver weights were observed between different groups of rats (Table S2). Comparable to human NAFLD patients, we observed that rats on an HFD developed insulin resistance (IR) [27], as shown by elevated plasma insulin levels with the HFD. Indeed, it has been shown that insulin resistance is commonly associated with obesity-induced hepatic steatosis [28,29]. Furthermore, hepatic lipid accumulation is known to activate intrahepatic inflammatory pathways, which stimulate the pro-inflammatory cytokine production, in turn leading to both hepatic and peripheral insulin resistance [30,31]. Upon administration of the extracellular CTSD inhibitor, we observed that HFD-fed rats showed a significant reduction in plasma insulin levels and improvement of steatosis, two important characteristics of diet-induced NAFLD. Hence, these data provide initial evidence that extracellular CTSD activity is involved with the regulation of lipid metabolism and thereby controls insulin sensitivity. However, the trend towards reduced food intake suggests additional potential effects on energy expenditure, which should be further investigated in the future. Additionally, though hepatic damage and inflammation were not increased in SD rats fed an HFD, treatment with the extracellular CTSD inhibitor showed reduced hepatic TNFα levels and AST/ALT ratio, indicating an improvement in hepatic damage and inflammation. Moreover, we validated this finding in primary macrophages and in an in vitro setup in which hepatic cells were incubated with a macrophage-derived conditioned medium, an approach that has been previously used to investigate crosstalk between hepatic immune and parenchymal cells [32,33]. As such, our findings suggest that macrophage-derived extracellular CTSD activity influences hepatic lipid metabolism and inflammation. However, despite the promising first results, the potential minor effects of intracellular CTSD and of the trend towards reduced food intake in the inhibitor-treated group suggest additional non-beneficial effects related to intracellular CTSD inhibition. To determine whether these non-beneficial metabolic effects are related directly to intracellular CTSD, a comparison with a compound with high permeability and low efflux (indicating that the compound mainly exerts its effects intracellularly) is necessary. Likewise, lower concentrations of the compound should be investigated in several NAFLD models before transition to the clinic is made.

Substantial evidence points towards the role of cathepsins in the context of NAFLD. Inactivation of cathepsin B in a dietary murine model of NAFLD prevented the development of hepatic steatosis [34]. Additionally, cathepsin B was found to be involved in hepatic injury and in progression of liver fibrosis [35,36]. Similarly, we have shown that lysosomal cholesterol accumulation inside Kupffer cells of *Ldlr-/-* mice leads to increased CTSD activity in the liver [37]. CTSD is a key lysosomal protease that affects many fundamental functions in the cell. A reduction in cellular CTSD expression or catalytic activity leads to devastating neurodegenerative disorders [38,39]. In contrast, an increase in extracellular CTSD expression and activity is associated with many types of cancers [40,41,42]. Owing to this differential expression and secretion of CTSD in many pathophysiological processes, there is increased awareness for the extracellular fraction of CTSD to play a role in health and disease. However, to date, research primarily focuses on targeting the whole fraction of CTSD [43], which is known to disturb physiological processes, resulting in serious side effects [44]. Hence, in the present study, we specifically investigated the benefits of targeting the extracellular fraction of CTSD in the context of NAFLD. 

Though the mechanisms underlying the secretion of CTSD are not yet known, changes in the lysosomal pH or lysosomal accumulation of poorly degradable lipids are known to cause mistargeting of CTSD into the extracellular milieu [45,46]. Additionally, cholesterol-filled lysosomes have been shown to induce disturbances in the lysosomal enzyme trafficking pathway that can potentially lead to increased levels of lysosomal enzymes in the plasma [47,48]. In vitro, particularly, the intracellular accumulation of the oxidized fraction of cholesterol has been shown to enhance extracellular secretion of CTSD [49,50]. Building further on this knowledge, we found in this study that targeting the activity of the extracellular fraction of CTSD in oxLDL-loaded macrophages improves inflammation and lipid metabolism, suggesting that macrophage-derived extracellular CTSD has a key role in lipid metabolism and inflammation.

Our observation that extracellular CTSD activity is involved with the pathogenesis of NAFLD implies that CTSD remains active in the circulation. Indeed, modulating the vacuolar type H+ ATPase pump by macrophages creates a more acidic pericellular space, in which lysosomal enzymes such as CTSD can remain active [51]. Moreover, several findings have implicated that upon secretion, anchorage of CTSD to the cell surface ameliorates its ability to interact with specific extracellular substrates [52]. The combination of an acidic pericellular environment and localization proximal to the cell proposes a potential mechanism by which extracellular CTSD activity can influence physiological processes. However, the question remains of which underlying mechanism is responsible for the observed improvements in lipid metabolism and inflammation upon inhibition of extracellular CTSD activity. In the current study, extracellular inhibition of CTSD upregulated the expression of *Cyp27a1* in macrophages. *Cyp27a1* is a gene encoding for an essential enzyme responsible for regulating cholesterol, fatty acid and bile acid metabolism through modulation of the mitochondrial P450 enzyme sterol 27-hydroxylase. In the liver, sterol 27-hydroxylase catalyzes the first step of the alternative bile acid biosynthetic pathway from cholesterol [53]. Previously, we have demonstrated the potential of *Cyp27a1* to modulate intracellular cholesterol distribution in Kupffer cells [37]. Of note, *Cyp27a1* has been shown to regulate ATP-binding cassette transporter A1 (ABCA1), an exporter of cellular lipids [54]. Based on this evidence, a possible explanation for our current findings is that the extracellular CTSD binds to one of the cell surface lipid transporters such as ABCA1 or scavenger receptors, which in turn blocks cholesterol transport, preventing regulation of cellular cholesterol and efflux pathways. It is therefore feasible that CTSD acts as an extracellular messenger interacting with an as yet unidentified cell surface receptor and regulates lipid homeostasis in addition to inflammation. In this regard, extracellular CTSD has recently been shown to be involved with ABCA1 regulation via influencing the low-density lipoprotein receptor-related protein 1 (LRP1) [55]. Additionally, we here observed differential expression of *Cd36* and *Sr-a* upon extracellular CTSD inhibition, suggesting an involvement of these scavenger receptors. Moreover, modulating proteases is known to influence the transcriptional regulation of cholesterol handling via *Srebp* [56] and *Nfe2l1* [57]. However, further studies are warranted to decipher the exact underlying mechanism of extracellular CTSD function.

In conclusion, we provide for the first time evidence that extracellular CTSD has a central role in the progression of NAFLD. In contrast to conventional therapeutic targeting of cathepsins, our data demonstrate that inhibiting specifically the extracellular fraction of CTSD can be a valuable therapeutic strategy for NAFLD. Further studies that investigate the downstream targets regulated by extracellular CTSD will provide deeper understanding of the mechanisms of NAFLD pathogenesis.

## Supplementary Materials

The following are available online at https://www.mdpi.com/2218-273X/9/5/171/s1, **Figure S1:** Inhibitory activity of CTD-002 on CTSD activity. **Figure S2:** Schematic representation of the in vivo setup. **Figure S3**: Effect of CTD-002 in bone marrow-derived macrophages under control conditions. **Figure S4:** *Cd36* gene expression levels of BMDMs and HepG2 cells. **Figure S5:** Food consumption of Sprague–Dawley rats. **Figure S6:** Representative images of fat droplets stained by haematoxylin and eosin. **Figure S7:** Impact of high-fat diet on hepatic fat deposition in Sprague–Dawley rats. Upper panels, Chromotrope Aniline Blue staining; bottom panels, Sirius Red staining. **Table S1:** Composition of low and high fat diets. **Table S2**: Relative liver weight (per 100 g of body weight) of the experimental groups of rats.

## Abbreviations

NAFLD	non-alcoholic fatty liver disease
NASH	non-alcoholic steatohepatitis
CTSD	cathepsin D
HFD	high-fat diet
LFD	low-fat diet
BMDMs	Bone Marrow-derived macrophages
oxLDL	oxidized low-density lipoprotein
Wt	wildtype
LDL(R)	low-density lipoprotein (receptor)
SD	Sprague–Dawley
ELISA	enzyme-linked immune sorbent assay
LCM	L929-conditioned medium
TNF	tumor necrosis factor
LPS	lipopolysaccharide
AST	aspartate transaminase
ALT	alanine transaminase
MetS	metabolic syndrome
Pep-A	Pepstatin-A

## References

[1] Younossi Z.M., Koenig A.B., Abdelatif D., Fazel Y., Henry L., Wymer M. (2016). Global epidemiology of nonalcoholic fatty liver disease-Meta-analytic assessment of prevalence, incidence, and outcomes. Hepatology.

[2] Tandra S., Yeh M.M., Brunt E.M., Vuppalanchi R., Cummings O.W., Unalp-Arida A., Wilson L.A., Chalasani N. (2011). Presence and significance of microvesicular steatosis in nonalcoholic fatty liver disease. J. Hepatol..

[3] Takahashi Y., Fukusato T. (2014). Histopathology of nonalcoholic fatty liver disease/nonalcoholic steatohepatitis. World J. Gastroenterol..

[4] Contos M.J., Choudhury J., Mills A.S., Sanyal A.J. (2004). The histologic spectrum of nonalcoholic fatty liver disease. Clin. Liver Dis..

[5] Musso G., Cassader M., Rosina F., Gambino R. (2012). Impact of current treatments on liver disease, glucose metabolism and cardiovascular risk in non-alcoholic fatty liver disease (NAFLD): A systematic review and meta-analysis of randomised trials. Diabetologia.

[6] Cheung O., Sanyal A.J. (2008). Abnormalities of lipid metabolism in nonalcoholic fatty liver disease. Semin. Liver Dis..

[7] Lindstedt L., Lee M., Oorni K., Bromme D., Kovanen P.T. (2003). Cathepsins F and S block HDL3-induced cholesterol efflux from macrophage foam cells. Biochem. Biophys. Res. Commun..

[8] Tertov V.V., Orekhov A.N. (1997). Metabolism of native and naturally occurring multiple modified low-density lipoprotein in smooth muscle cells of human aortic intima. Exp. Mol. Pathol..

[9] Thelen A.M., Zoncu R. (2017). Emerging Roles for the Lysosome in Lipid Metabolism. Trends Cell Biol..

[10] Lutgens S.P.M., Cleutjens K.B.J.M., Daemen M.J.A.P., Heeneman S. (2007). Cathepsin cysteine proteases in cardiovascular disease. FASEB J..

[11] Benes P., Vetvicka V., Fusek M. (2008). Cathepsin D—Many functions of one aspartic protease. Crit. Rev. Oncol. Hematol..

[12] Moallem S.A., Nazemian F., Eliasi S., Alamdaran S.A., Shamsara J., Mohammadpour A.H. (2011). Correlation between cathepsin D serum concentration and carotid intima-media thickness in hemodialysis patients. Int. Urol. Nephrol..

[13] Snir J.A., Suchy M., St Lawrence K., Hudson R.H.E., Pasternak S.H., Bartha R. (2015). Prolonged In Vivo Retention of a Cathepsin D Targeted Optical Contrast Agent in a Mouse Model of Alzheimer’s Disease. J. Alzheimers Dis..

[14] Briozzo P., Badet J., Capony F., Pieri I., Montcourrier P., Barritault D., Rochefort H. (1991). MCF7 mammary cancer cells respond to bFGF and internalize it following its release from extracellular matrix: A permissive role of cathepsin D. Exp. Cell Res..

[15] Porter K., Lin Y.Z., Liton P.B. (2013). Cathepsin B Is Up-Regulated and Mediates Extracellular Matrix Degradation in Trabecular Meshwork Cells Following Phagocytic Challenge. PLoS ONE.

[16] Naseem R.H., Hedegard W., Henry T.D., Lessard J., Sutter K., Katz S.A. (2005). Plasma cathepsin D isoforms and their active metabolites increase after myocardial infarction and contribute to plasma renin activity. Basic Res. Cardiol..

[17] Walenbergh S.M., Houben T., Hendrikx T., Jeurissen M.L., van Gorp P.J., Vreugdenhil A.C., Adriaanse M.P., Buurman W.A., Hofker M.H., Mosca A. (2015). Plasma cathepsin D levels: A novel tool to predict pediatric hepatic inflammation. Am. J. Gastroenterol..

[18] Walenbergh S.M., Houben T., Rensen S.S., Bieghs V., Hendrikx T., van Gorp P.J., Oligschlaeger Y., Jeurissen M.L., Gijbels M.J., Buurman W.A. (2016). Plasma cathepsin D correlates with histological classifications of fatty liver disease in adults and responds to intervention. Sci. Rep..

[19] Houben T., Oligschlaeger Y., Hendrikx T., Bitorina A.V., Walenbergh S.M.A., van Gorp P.J., Gijbels M.J.J., Friedrichs S., Plat J., Schaap F.G. (2017). Cathepsin D regulates lipid metabolism in murine steatohepatitis. Sci. Rep..

[20] Marciniszyn J., Hartsuck J.A., Tang J. (1976). Mode of inhibition of acid proteases by pepstatin. J. Biol. Chem..

[21] Lieber C.S., Leo M.A., Mak K.M., Xu Y., Cao Q., Ren C., Ponomarenko A., DeCarli L.M. (2004). Model of nonalcoholic steatohepatitis. Am. J. Clin. Nutr..

[22] Dixon S.L., Smondyrev A.M., Knoll E.H., Rao S.N., Shaw D.E., Friesner R.A. (2006). PHASE: A new engine for pharmacophore perception, 3D QSAR model development, and 3D database screening: 1. Methodology and preliminary results. J. Comput. Aided Mol. Des..

[23] Dixon S.L., Smondyrev A.M., Rao S.N. (2006). PHASE: A novel approach to pharmacophore modeling and 3D database searching. Chem. Biol. Drug Des..

[24] Friesner R.A., Banks J.L., Murphy R.B., Halgren T.A., Klicic J.J., Mainz D.T., Repasky M.P., Knoll E.H., Shelley M., Perry J.K. (2004). Glide: A new approach for rapid, accurate docking and scoring. 1. Method and assessment of docking accuracy. J. Med. Chem..

[25] Halgren T.A., Murphy R.B., Friesner R.A., Beard H.S., Frye L.L., Pollard W.T., Banks J.L. (2004). Glide: A new approach for rapid, accurate docking and scoring. 2. Enrichment factors in database screening. J. Med. Chem..

[26] Ahmed U., Redgrave T.G., Oates P.S. (2009). Effect of dietary fat to produce non-alcoholic fatty liver in the rat. J. Gastroenterol. Hepatol..

[27] Zou Y., Li J., Lu C., Wang J., Ge J., Huang Y., Zhang L., Wang Y. (2006). High-fat emulsion-induced rat model of nonalcoholic steatohepatitis. Life Sci..

[28] Kitade H., Chen G., Ni Y., Ota T. (2017). Nonalcoholic Fatty Liver Disease and Insulin Resistance: New Insights and Potential New Treatments. Nutrients.

[29] Perry R.J., Samuel V.T., Petersen K.F., Shulman G.I. (2014). The role of hepatic lipids in hepatic insulin resistance and type 2 diabetes. Nature.

[30] Samuel V.T., Liu Z.X., Qu X.Q., Elder B.D., Bilz S., Befroy D., Romanelli A.J., Shulman G.I. (2004). Mechanism of hepatic insulin resistance in non-alcoholic fatty liver disease. J. Biol. Chem..

[31] Yamaguchi K., Nishimura T., Ishiba H., Seko Y., Okajima A., Fujii H., Tochiki N., Umemura A., Moriguchi M., Sumida Y. (2015). Blockade of interleukin 6 signalling ameliorates systemic insulin resistance through upregulation of glucose uptake in skeletal muscle and improves hepatic steatosis in high-fat diet fed mice. Liver Int..

[32] Pardo V., Gonzalez-Rodriguez A., Guijas C., Balsinde J., Valverde A.M. (2015). Opposite cross-talk by oleate and palmitate on insulin signaling in hepatocytes through macrophage activation. J. Biol. Chem..

[33] Melino M., Gadd V.L., Walker G.V., Skoien R., Barrie H.D., Jothimani D., Horsfall L., Jones A., Sweet M.J., Thomas G.P. (2012). Macrophage secretory products induce an inflammatory phenotype in hepatocytes. World J. Gastroenterol..

[34] Feldstein A.E., Werneburg N.W., Canbay A., Guicciardi M.E., Bronk S.F., Rydzewski R., Burgart L.J., Gores G.J. (2004). Free fatty acids promote hepatic lipotoxicity by stimulating TNF-alpha expression via a lysosomal pathway. Hepatology.

[35] Li Z.Z., Berk M., McIntyre T.M., Gores G.J., Feldstein A.E. (2008). The lysosomal-mitochondrial axis in free fatty acid-induced hepatic lipotoxicity. Hepatology.

[36] Moles A., Tarrats N., Fernandez-Checa J.C., Mari M. (2009). Cathepsins B and D Drive Hepatic Stellate Cell Proliferation and Promote Their Fibrogenic Potential. Hepatology.

[37] Bieghs V., Hendrikx T., van Gorp P.J., Verheyen F., Guichot Y.D., Walenbergh S.M.A., Jeurissen M.L.J., Gijbels M., Rensen S.S., Bast A. (2013). The Cholesterol Derivative 27-Hydroxycholesterol Reduces Steatohepatitis in Mice. Gastroenterology.

[38] Saftig P., Hetman M., Schmahl W., Weber K., Heine L., Mossmann H., Koster A., Hess B., Evers M., von Figura K. (1995). Mice deficient for the lysosomal proteinase cathepsin D exhibit progressive atrophy of the intestinal mucosa and profound destruction of lymphoid cells. EMBO J..

[39] Koike M., Nakanishi H., Saftig P., Ezaki J., Isahara K., Ohsawa Y., Schulz-Schaeffer W., Watanabe T., Waguri S., Kametaka S. (2000). Cathepsin D deficiency induces lysosomal storage with ceroid lipofuscin in mouse CNS neurons. J. Neurosci..

[40] Glondu M., Coopman P., Laurent-Matha V., Garcia M., Rochefort H., Liaudet-Coopman E. (2001). A mutated cathepsin-D devoid of its catalytic activity stimulates the growth of cancer cells. Oncogene.

[41] Vetvicka V., Vetvickova J., Fusek M. (1998). Effect of procathepsin D and its activation peptide on prostate cancer cells. Cancer Lett..

[42] Vetvicka V., Vetvickova J., Benes P. (2004). Role of enzymatically inactive procathepsin D in lung cancer. Anticancer Res..

[43] Dubey V., Luqman S. (2017). Cathepsin D as a Promising Target for the Discovery of Novel Anticancer Agents. Curr. Cancer Drug Targets.

[44] Ruan H., Hao S., Young P., Zhang H. (2015). Targeting Cathepsin B for Cancer Therapies. Horiz. Cancer Res..

[45] Settembre C., Di Malta C., Polito V.A., Garcia Arencibia M., Vetrini F., Erdin S., Erdin S.U., Huynh T., Medina D., Colella P. (2011). TFEB links autophagy to lysosomal biogenesis. Science.

[46] Rosenfeld M.G., Kreibich G., Popov D., Kato K., Sabatini D.D. (1982). Biosynthesis of lysosomal hydrolases: Their synthesis in bound polysomes and the role of co- and post-translational processing in determining their subcellular distribution. J. Cell Biol..

[47] Ungewickell A.J., Majerus P.W. (1999). Increased levels of plasma lysosomal enzymes in patients with Lowe syndrome. Proc. Natl. Acad. Sci. USA.

[48] Hultberg B., Isaksson A., Sjoblad S., Ockerman P.A. (1980). Acid hydrolases in serum from patients with lysosomal disorders. Clin. Chim. Acta.

[49] Hoppe G., O’Neil J., Hoff H.F., Sears J. (2004). Products of lipid peroxidation induce missorting of the principal lysosomal protease in retinal pigment epithelium. BBA-Mol. Basis Dis..

[50] Li W., Yuan X.M., Olsson A.G., Brunk U.T. (1998). Uptake of oxidized LDL by macrophages results in partial lysosomal enzyme inactivation and relocation. Arterioscler. Thromb. Vasc. Biol..

[51] Reddy V.Y., Zhang Q.Y., Weiss S.J. (1995). Pericellular mobilization of the tissue-destructive cysteine proteinases, cathepsins B, L, and S, by human monocyte-derived macrophages. Proc. Natl. Acad. Sci. USA.

[52] Neurath H. (1984). Evolution of proteolytic enzymes. Science.

[53] Dubrac S., Lear S.R., Ananthanarayanan M., Balasubramaniyan N., Bollineni J., Shefer S., Hyogo H., Cohen D.E., Blanche P.J., Krauss R.M. (2005). Role of CYP27A in cholesterol and bile acid metabolism. J. Lipid Res..

[54] Oram J.F. (2003). HDL apolipoproteins and ABCA1: Partners in the removal of excess cellular cholesterol. Arterioscler. Thromb. Vasc. Biol..

[55] Oldoni F., van Capelleveen J.C., Dalila N., Wolters J.C., Heeren J., Sinke R.J., Hui D.Y., Dallinga-Thie G.M., Frikke-Schmidt R., Hovingh K.G. (2018). Naturally Occurring Variants in LRP1 (Low-Density Lipoprotein Receptor-Related Protein 1) Affect HDL (High-Density Lipoprotein) Metabolism Through ABCA1 (ATP-Binding Cassette A1) and SR-B1 (Scavenger Receptor Class B Type 1) in Humans. Arterioscler. Thromb. Vasc. Biol..

[56] Brown M.S., Goldstein J.L. (1997). The SREBP pathway: Regulation of cholesterol metabolism by proteolysis of a membrane-bound transcription factor. Cell.

[57] Widenmaier S.B., Snyder N.A., Nguyen T.B., Arduini A., Lee G.Y., Arruda A.P., Saksi J., Bartelt A., Hotamisligil G.S. (2017). NRF1 Is an ER Membrane Sensor that Is Central to Cholesterol Homeostasis. Cell.

