# Supplementary material for: Inhibiting Extracellular Cathepsin D Reduces Hepatic Steatosis in Sprague–Dawley Rats
Source: Biomolecules. 2019 May 4;9(5):171. doi: 10.3390/biom9050171 (PMC6571693; doi:10.3390/biom9050171)
Supplement: Supplementary file 1 [file biomolecules-09-00171-s001.pdf]

Figure S1: Inhibitory activity of CTD-002 on CTSD activity

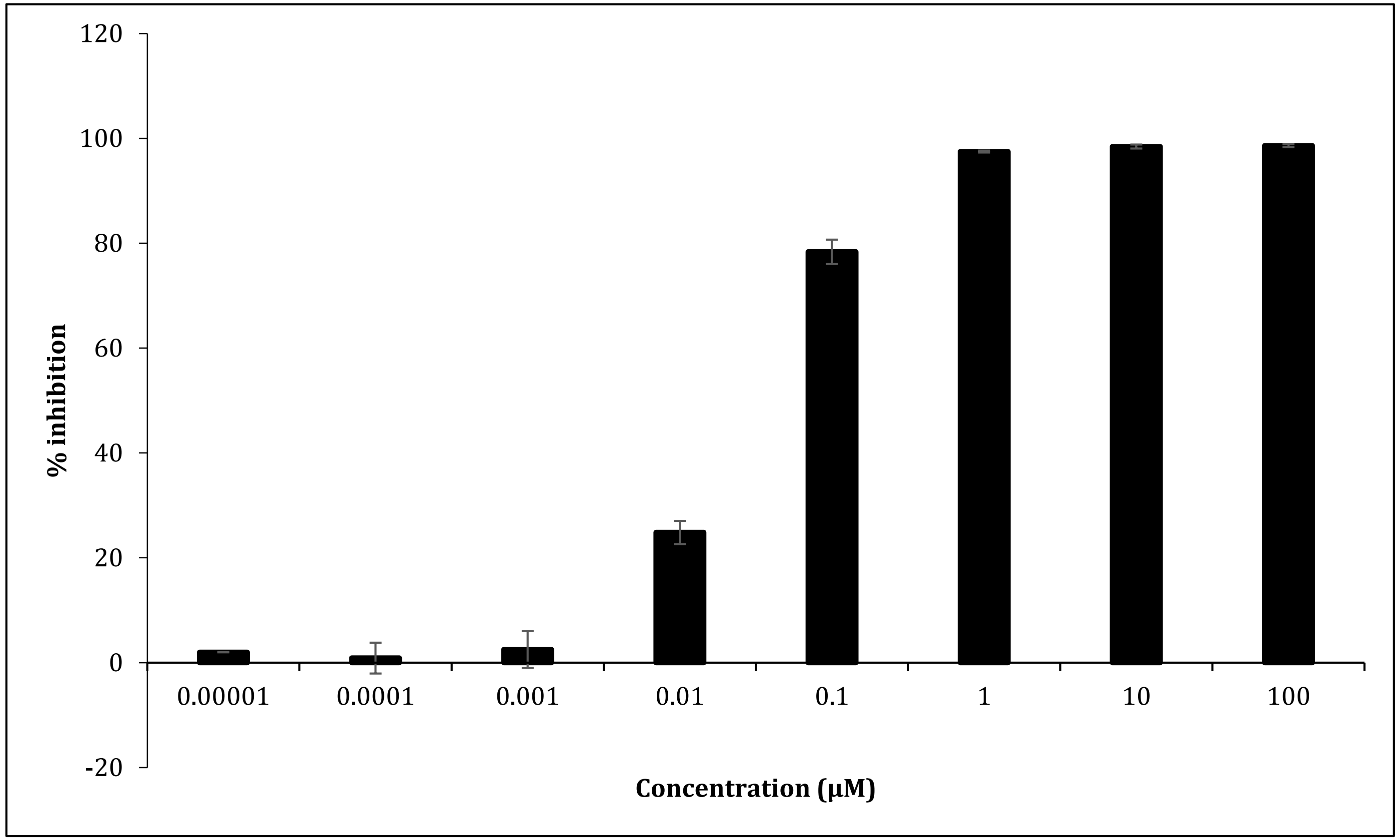

Figure S2: Schematic representation of the *in-vivo* setup

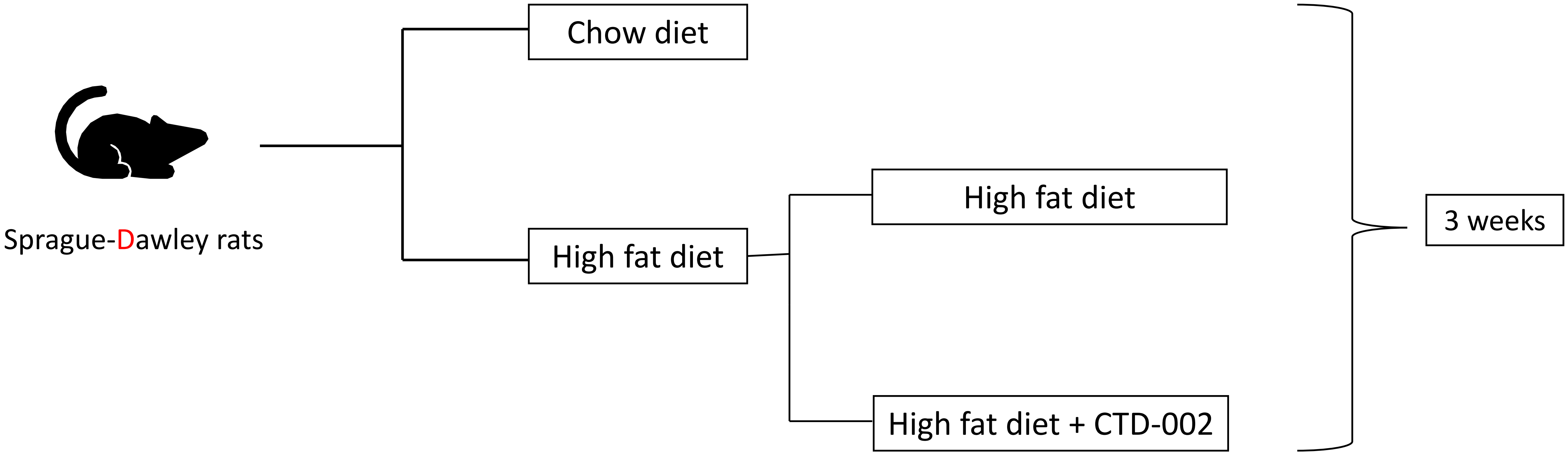

Figure S3: Effect of CTD-002 in bone marrow-derived macrophages under control conditions

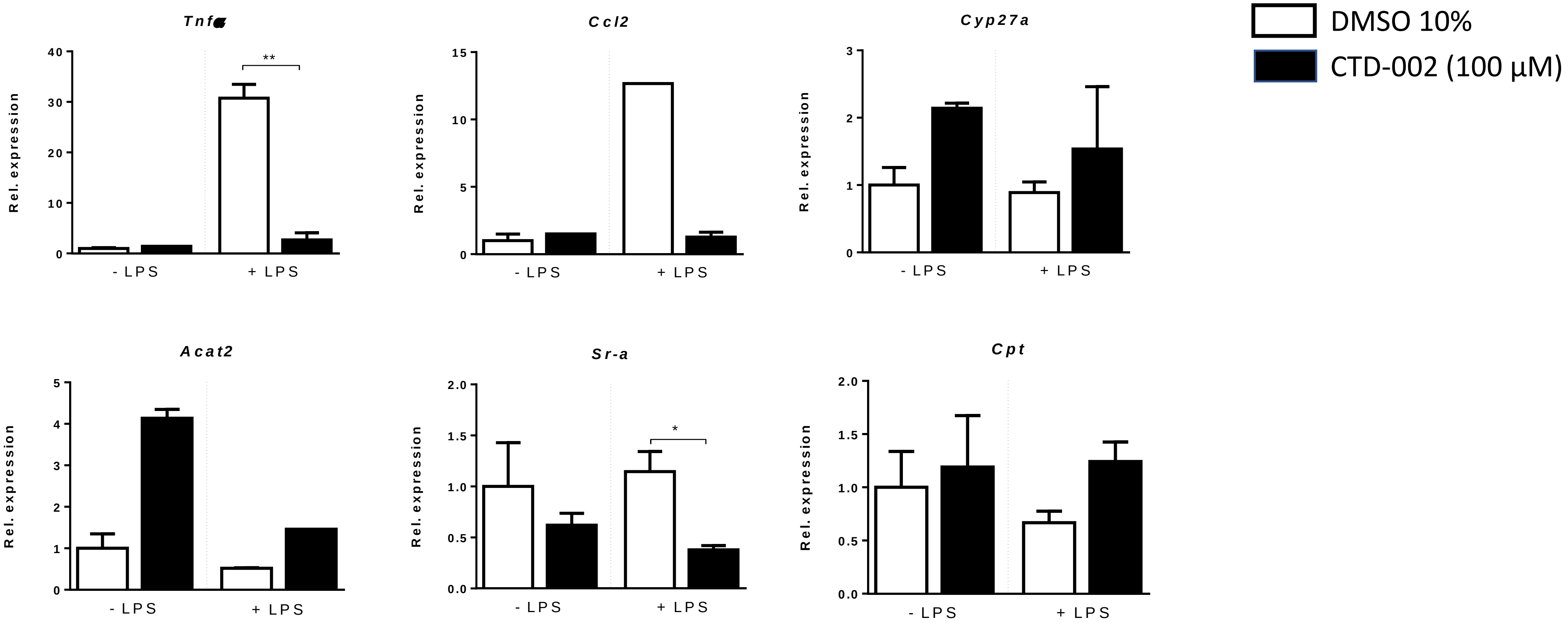

Figure S4: *Cd36* gene expression levels of BMDMs and HepG2

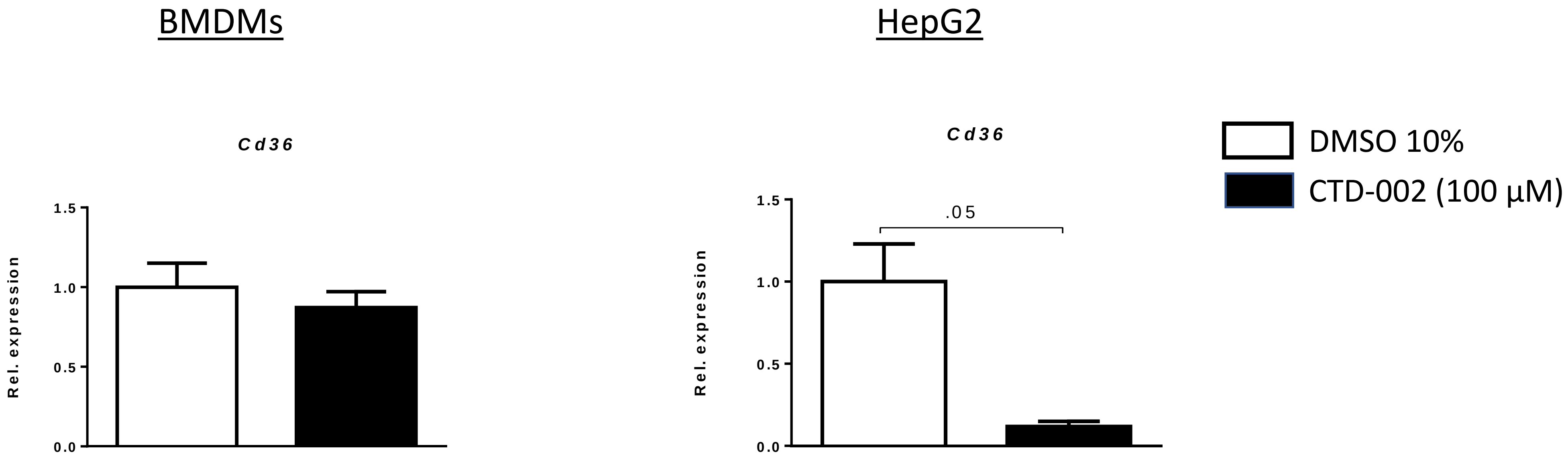

Figure S5: Food consumption of Sprague-Dawley rats

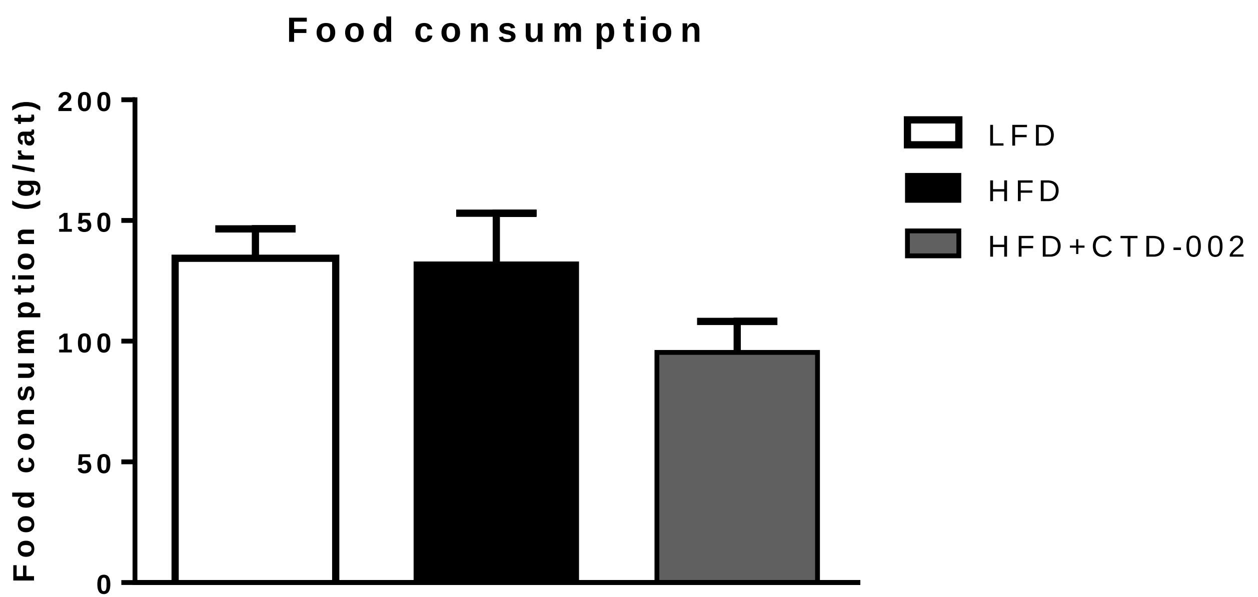

Figure S6: Representative images of fat droplets stained by haematoxylin and eosin staining

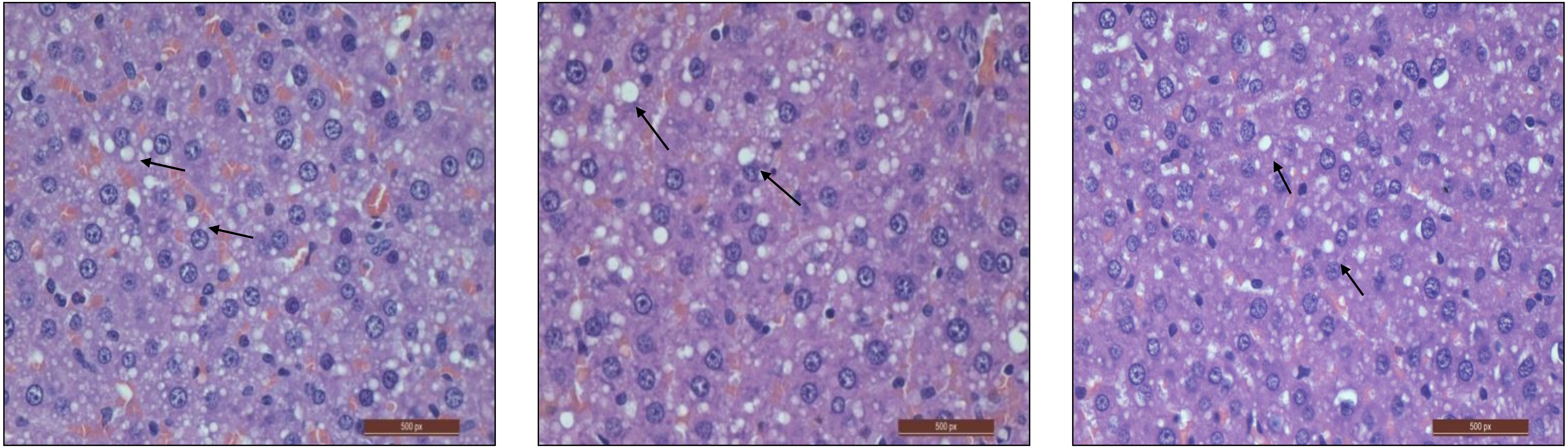

Low fat diet High fat diet High fat diet + CTD-002

Figure S7: Impact of high-fat diet on hepatic fat deposition in Sprague-Dawley rats

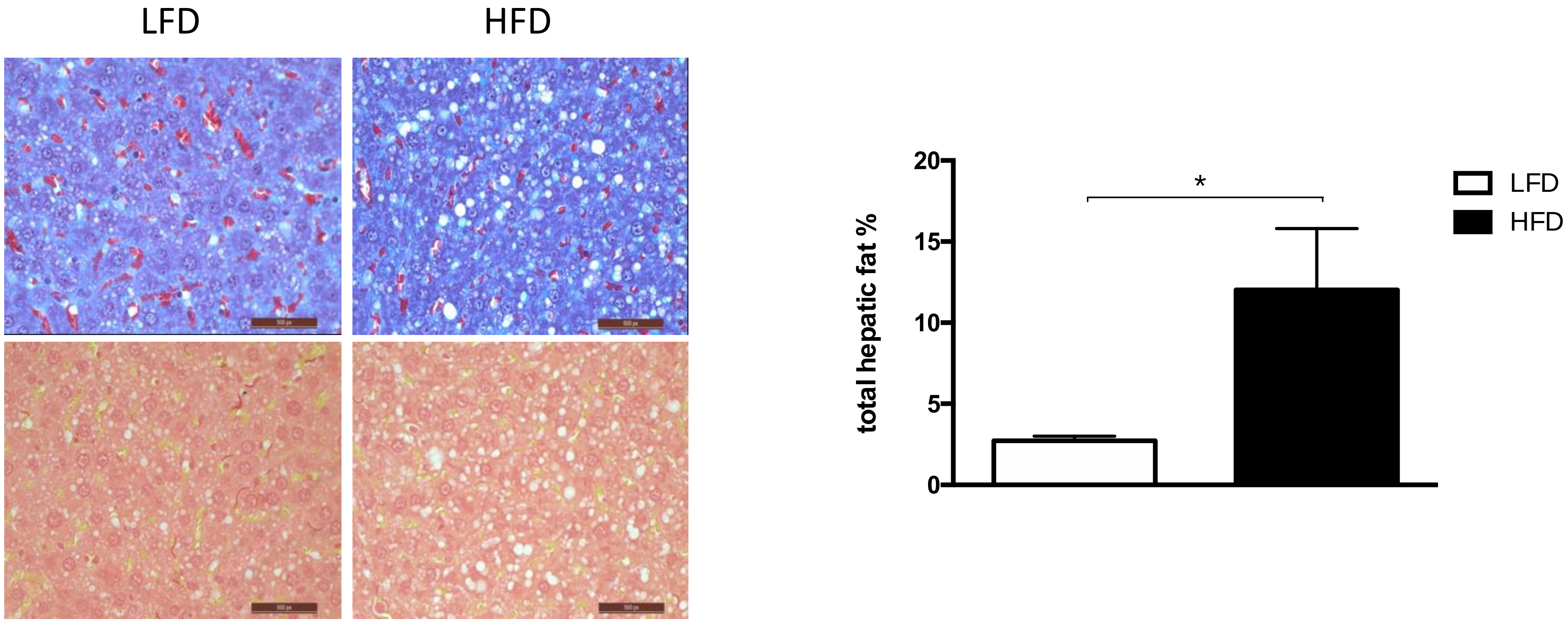

Table S1: Composition of low and high fat diets

Ingredients	Low fat diet (gm)	High fat diet (gm)
Casein	200	200
L-Cystine	3	3
Corn starch	506	0
Maltodextrin 10	125	125
Sucrose	68.8	68.8
Cellulose	50	50
Soybean Oil	25	25
Lard*	20	245
DiCalcium Phosphate	13	13
Calcium Carbonate	5.5	5.5
Choline Bitartrate	2	2
Potassium Citrate	16.5	16.5
Mineral Mix	10	10
Vitamin Mix	10	10
FD&C Blue Dye	0.01	0.05
FD&C yellow Dye	0.04	0

*Typical analysis of cholesterol in lard = 0.72 mg/gram.

Table S2**:** Relative liver weight (per 100 g of body weight) of the experimental groups of rats

Group	Liver weight mg/100g body weight (Mean ± SD)
LFD	3.67 ± 0.61
HFD	3.73 ± 0.27
HFD + CTD-002	3.73 ± 0.47
